# The mechanics of malaria parasite invasion of the human erythrocyte – towards a reassessment of the host cell contribution

**DOI:** 10.1111/cmi.12557

**Published:** 2016-01-11

**Authors:** Marion Koch, Jake Baum

**Affiliations:** ^1^ Department of Life SciencesImperial College LondonSir Alexander Fleming Building, Exhibition RoadSouth KensingtonLondonSW7 2AZUK

## Abstract

Despite decades of research, we still know little about the mechanics of *Plasmodium* host cell invasion. Fundamentally, while the essential or non‐essential nature of different parasite proteins is becoming clearer, their actual function and how each comes together to govern invasion are poorly understood. Furthermore, in recent years an emerging world view is shifting focus away from the parasite actin–myosin motor being the sole force responsible for entry to an appreciation of host cell dynamics and forces and their contribution to the process. In this review, we discuss merozoite invasion of the erythrocyte, focusing on the complex set of pre‐invasion events and how these might prime the red cell to facilitate invasion. While traditionally parasite interactions at this stage have been viewed simplistically as mediating adhesion only, recent work makes it apparent that by interacting with a number of host receptors and signalling pathways, combined with secretion of parasite‐derived lipid material, that the merozoite may initiate cytoskeletal re‐arrangements and biophysical changes in the erythrocyte that greatly reduce energy barriers for entry. Seen in this light *Plasmodium* invasion may well turn out to be a balance between host and parasite forces, much like that of other pathogen infection mechanisms.

## Introduction

Systems biology has entirely transformed our understanding of the malaria parasites, protozoan pathogens from the genus *Plasmodium* (Le Roch *et al*., 2012). Published genomes for the core *Plasmodium* species that infect humans and rodents have given us a blueprint for gene discovery, while transcriptome and proteomic studies have substantially mapped out the active genetic elements across parasite lifecycle stages. Most recently, with the adaptation of the state‐of‐the art technologies in genetics, e.g. the CRISPR/cas‐9 system, and structural biology, such as cryo‐electron microscopy, there has never been a more powerful time to interrogate the complex biology of these ancient pathogens. However, despite these incredible advances, we still know very little about what many parasite proteins actually (i.e. mechanistically) do.

Nowhere is the paucity of mechanistic understanding more keenly demonstrated than in the process of erythrocyte invasion by the blood stage merozoite. This process sets in motion all symptoms associated with malaria disease (White *et al*., 2014), and, unsurprisingly, many of the dominant merozoite proteins are some of the most advanced targets for vaccine development. This list includes merozoite surface and secreted proteins, among the earliest *Plasmodium* proteins described in any molecular detail (Peterson *et al*., 1989; Blackman *et al*., 1990). And yet, for nearly all of them, whether surface bound or secreted, we have very little insight into their precise function beyond an essential or non‐essential nature or a putative host cell binding partner. Recent advances with conditional‐knockout systems are certainly starting to readdress this imbalance (Yap *et al*., 2014; Das *et al*., 2015), but it is still early days. To be clear, even in the absence of a definitive assignment of function, there is a growing database of knowledge relating to the expression timing, structure and protein–protein interactions for core invasion effectors with much of it reviewed extensively in recent years (e.g. (Cowman *et al*., 2012; Paul *et al*., 2015)). Here, rather than repeat a systematic review of these proteins, our aim is to focus attention on the mechanics of the process, towards a functional framework that highlights where critical gaps in our knowledge exist, gaps that must be filled if we are to fully understand how merozoites *actually* enter the erythrocyte.

## Plasmodium host cell invasion – a black sheep or a conventional eukaryote?

**Figure 1:**
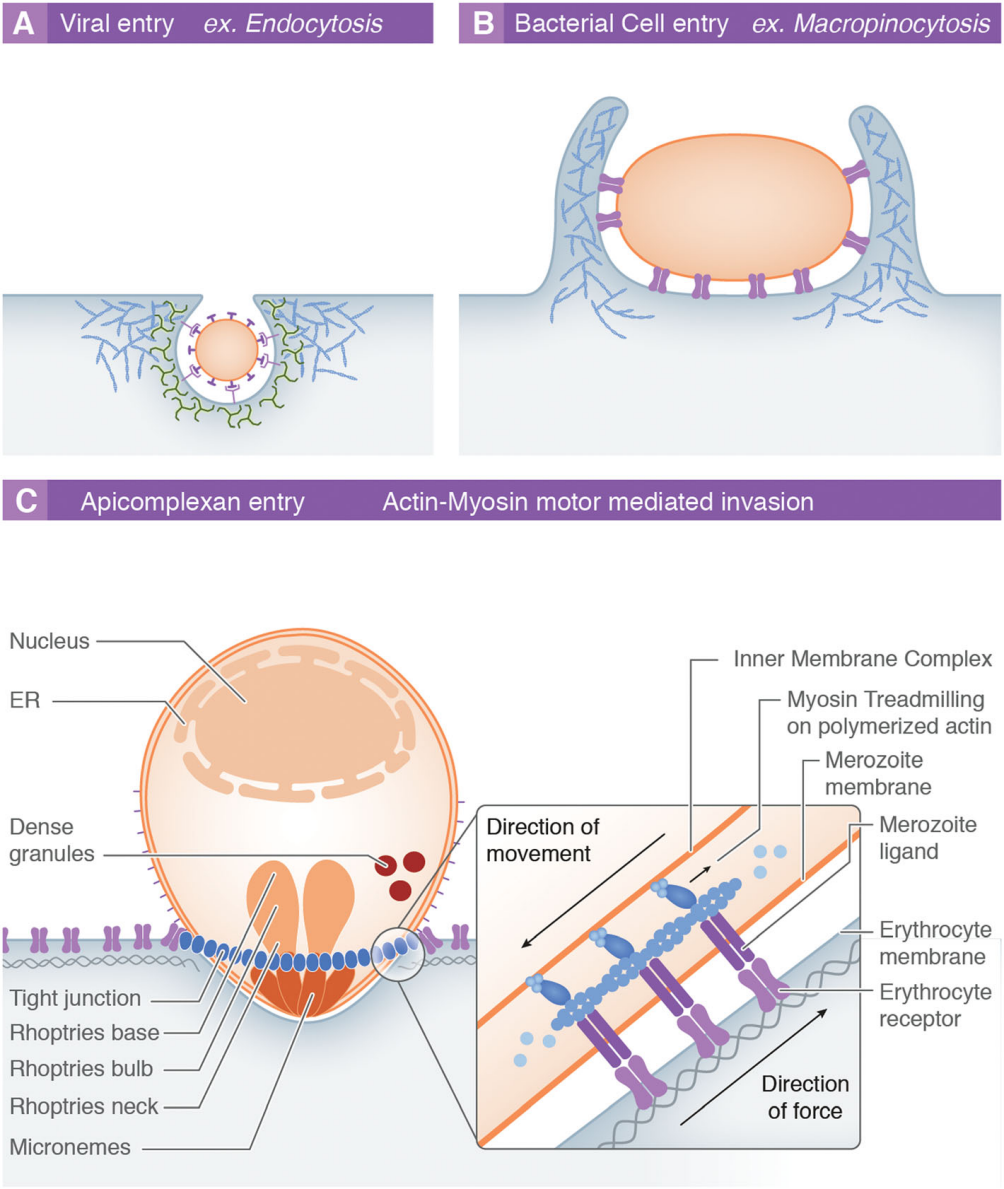
Mechanisms of host cell entry by intracellular pathogens. Most intracellular pathogens exploit host processes to overcome the intrinsic barriers to cell entry: the host cell membrane and cytoskeleton. A. The endocytic pathway, which regulates a range of cellular functions via controlled receptor internalization and recycling, is exploited by numerous viruses (e.g. rabies virus) to facilitate their own uptake. The process involves the recruitment and subversion of various host proteins (green), which mediate membrane curvature and vesicular scission. Host cell actin (red) polymerization is also required during the internalization process. B. Larger pathogens such as many intracellular bacteria (e.g. *Salmonella* spp.), protozoa and also certain viruses enter host cells through the initiation of host cell actin‐mediated membrane ruffles that fold over and lead to engulfment of cargo into large intracellular vesicles (macropinosomes). C. The model for apicomplexan cell entry involves active invasion using an actin–myosin motor. This involves myosin treadmilling on polymerized actin connected to apicomplexan surface ligands, which causes an inward movement of the pathogen without a response or involvement of the host cell. ER, endoplasmic reticulum.

Most intracellular pathogens invade target cells by manipulating host‐dependent signalling pathways that facilitate entry via membrane invagination, leading to the formation of a specialized entry vesicle that would otherwise function in endocytosis. This can involve several processes including formation of caveolae, clathrin cages, or the initiation of membrane extensions that literally engulf cargo such as during viral or bacterial entry (Fig. 1A and B) (de Souza Santos and Orth, 2015; Newsome and Marzook, 2015). Targeting the endocytosis pathway leads to the bending of the host cell membrane, a process heavily governed by physical forces derived from the active host cell cytoskeleton combined with high‐affinity receptor–ligand interactions between the pathogen surface and host receptors (e.g. (Pelkmans *et al*., 2002)). Where receptor–ligand adhesion is greater than the cost of membrane bending, i.e. akin to a Velcro^®^ sheet engulfing a tennis ball, the adhesion strength will drive host membrane deformation around the incoming pathogen, maximizing the area of ligand–receptor contact (Dasgupta *et al*., 2014a). In addition to receptor–ligand affinity, membrane wrapping will also depend on receptor–ligand density as well as more general host cell membrane physical properties. The latter includes the membrane's bending modulus (the intrinsic energy required for it to bend) or more broadly cytoskeletal (or tension) properties (Gefen, 2010). Thus, dissecting the mechanics of intracellular pathogen invasion requires a detailed appreciation of host cell factors as much as those of the pathogen.

Apicomplexan parasites, obligate intracellular protozoan pathogens, are thought to break these almost universal rules (Fig. 1C). This ancient phylum includes the etiological agents of toxoplasmosis, cryptosporidiosis and, the subject here, malaria. These parasites, unlike bacterial or viral infectious agents, are equipped with their own actin–myosin motor force that not only powers their motility (called gliding) but also provides the mechanical force to actively penetrate host cells (Heintzelman, 2015). This conserved system acts as a form of conveyor belt pushing the parasite into the host cell while receptor–ligand interactions (linked internally to the parasite motor) are driven rearwards. Much of our knowledge on apicomplexan invasion has come from studies on *Toxoplasma*, a parasite closely related to *Plasmodium*. Early studies with both parasites demonstrated that there was a reliance of invasion on actin–myosin dynamics (Miller *et al*., 1979; Dobrowolski and Sibley, 1996), dynamics presumed to be parasite derived. This led to a prevailing dogma wherein apicomplexan host cell entry differs from that via endocytosis, the host cell playing the role of passive bystander. The most influential of these studies showed that inhibiting the host cell cytoskeleton with low doses of the actin‐inhibitory drug cytochalasin D did not affect invasion efficiency by *Toxoplasma gondii* parasites (Dobrowolski and Sibley, 1996). Recently, this view has been challenged by microscopy evidence from both *Toxoplasma* tachyzoites and *Plasmodium* sporozoites (liver‐infective stage) where host cell cytoskeletal re‐arrangement and re‐organization can be clearly seen during invasion (Gonzalez *et al*., 2009). In addition, the advent of novel genetic engineering techniques recently made it possible to delete several of the key actin–myosin motor components (or presumed binding partners) in *T*. *gondii* challenging the predominance of the apicomplexan cell (Meissner *et al*., 2013, Egarter *et al*., 2014, Shen and Sibley, 2014). Although the importance of the actin–myosin motor driven force for efficient cell entry is indisputable, the relative contribution from the parasite motor is as a result receiving increased, although controversial, scrutiny (Meissner *et al*., 2013)

Despite this shift in world view, the erythrocyte, the target of the *Plasmodium* merozoite, is still widely regarded as a non‐dynamic cell that plays no role during invasion – extending the ‘passive host cell’ dogma. What is little appreciated is that this also runs contrary to extensive evidence that the erythrocyte membrane and its underlying cytoskeleton are very dynamic (Betz *et al*., 2009; Gokhin *et al*., 2015). Furthermore, there is evidence that it is manipulated during invasion by the merozoite (reviewed in (Zuccala and Baum, 2011)). When considered in this light, it is not unsurprising that genetic variants for erythrocyte cytoskeletal proteins, which give rise to changes in membrane stability, might give rise to markedly reduced *Plasmodium* invasion. For example, altering the spectrin oligomeric state *in vitro* strongly inhibits efficiency of human parasite *Plasmodium falciparum* invasion (Facer, 1995). Similarly, animal models carrying mutations in ankyrin, which causes abrogation of spectrin linkage to the cell membrane, show resistance to invasion by rodent *Plasmodium* species (Greth *et al*., 2012). Some of these effects may be specific to critical components of the erythrocyte cytoskeleton, but a significant portion may derive from simple biophysical requirements of the parasite for entry. Thus, a degree of host cell involvement, whether responsive or parasite mediated, in apicomplexan host cell invasion would seem to be inevitable, even for the red blood cell.

**Figure 2:**
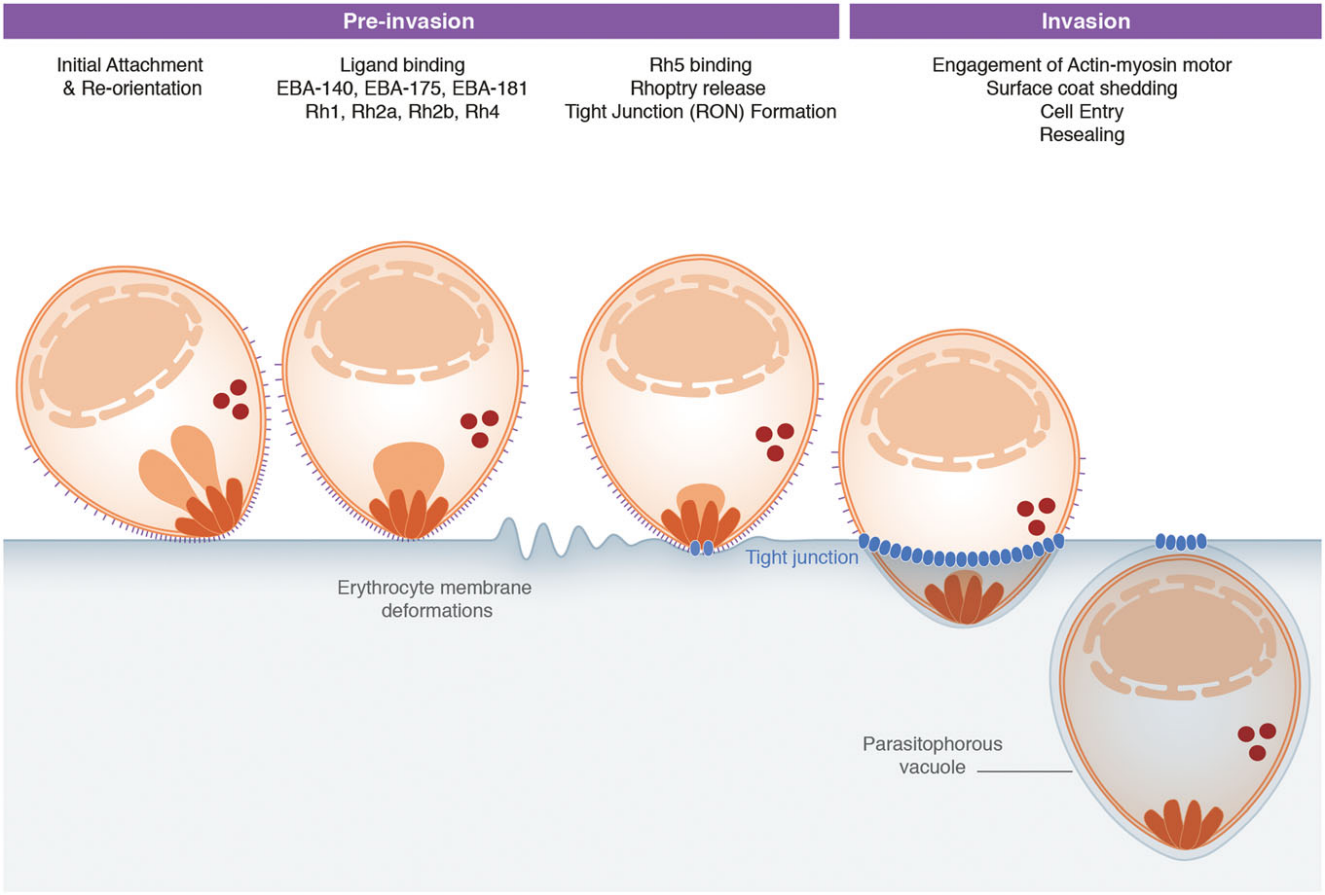
Overview of erythrocyte invasion by *Plasmodium falciparum* merozoites. Initial attachment of the merozoite to the erythrocyte can occur in any orientation. The merozoite then re‐orientates itself to position its apical pole in close contact with the host cell membrane. Numerous parasite host protein interactions occur at this point. Of particular interest are the interactions of EBA175 and Rh4 with their respective host receptors GPA and CR1, which appear to trigger erythrocyte responses visible as membrane deformations. Translocation of the RON complex (blue) across the erythrocyte membrane results in establishment of the tight junction, through which the merozoite enters the host cell into a parasitophorous vacuole utilizing force from its actin–myosin motor.

Erythrocyte invasion occurs through a complex multi‐stage process involving numerous parasite host protein interactions, with varying levels of redundancy (Paul *et al*., 2015), secretion of invasion‐associated parasite organelles and formation of the unique parasitophorous vacuole within which the parasite resides and develops (Lingelbach and Joiner, 1998). The rapid process is traditionally broken down into four distinct stages, defined by both live imaging and ultrastructural imaging studies: (i) initial merozoite attachment, (ii) re‐orientation of the merozoite to its apical end, (iii) formation of a tight or moving junction and (iv) complete invasion and sealing of the parasitophorous vacuole (see Fig. 2 for an overview).

## The early steps of invasion – committing to entry

Merozoite exit, otherwise called egress, from the previous cycle is itself a multi‐stage process that involves both processing of surface antigens and interactions with the host erythrocyte cytoskeleton (Bichet *et al*., 2014, Blackman and Carruthers, 2013; Das *et al*., 2015). On release, and following the chance encounter of a merozoite with a circulating red cell, initial attachment to the erythrocyte surface is likely supported by a family of merozoite surface proteins (MSPs), which coat the entire free parasite (Kadekoppala and Holder, 2010; Lin *et al*., 2014). MSP1 is the best characterised of these proteins as well as being the largest and most abundant (Blackman *et al*., 1990). Anchored via a glycosyl‐phosphatidylinositol modification, it is unlikely that MSP1 acts alone, rather it seems to form a platform for the assembly of a large protein complex containing numerous MSPs (including MSP6‐7 and MSPDBL1‐2) (Kadekoppala and Holder, 2010). While distinct regions of MSP1 have been shown capable of interacting with proteins on the red cell (e.g. Band 3 (Goel *et al*., 2003; Li *et al*., 2004) or glycophorin A (GPA) (Baldwin *et al*., 2015)), the function of the complex as a whole is far from clear. Blocking MSP1 (and other heparin binding (Boyle *et al*., 2010)) ligands renders the majority of merozoites unable to attach to erythrocytes, which adds weight to its functional role in invasion (Boyle *et al*., 2010). Supporting this further, the few merozoites that do attach do so with a reduction in attachment force of over 75% (Crick *et al*., 2014). However, beyond this evidence, and with substantial data now supporting a role in egress (Das *et al*., 2015), MSP involvement in the mechanics of entry is no longer a forgone conclusion. One thing that is certain is that at this stage, commitment to invasion has not occurred.

In contrast to the MSPs, which are surface bound from early on in parasite development, the parasite invasion proteins utilized during the next steps of pre‐invasion are secreted from the apical organelles, the micronemes and rhoptries (Fig. 2). Their respective constituents are believed to have a much greater affinity for receptors on the red cell surface and are likely released sequentially. The concept of organelle localisation determining the timing or release and functional classification is not new in apicomplexan biology (having been defined in *Toxoplasma* (Carruthers and Sibley, 1997)). However, what has been overlooked is the potential that there is much more sub‐compartmentalisation within both rhoptries and likely micronemes than is often appreciated (Richard *et al*., 2009; Zuccala *et al*., 2012). Ultrastructure within the rhoptries (neck, bulb and base) is particularly striking and has been characterized both in *Plasmodium* and *Toxoplasma* spp. (Bannister *et al*., 2000; Lemgruber *et al*., 2011). Although the staining appears to be technique dependent (Hanssen *et al*., 2013), there is, nevertheless, extensive evidence for a localized organization of rhoptry proteins within this organelle (Bannister *et al*., 2000; Bradley *et al*., 2005; Zuccala *et al*., 2012) that could relate to temporal release of constituent proteins. Thus, rhoptry neck secretion likely precedes rhoptry bulb or base secretion (Riglar *et al*., 2011; Zuccala *et al*., 2012). It is, however, important to note that not all rhoptry proteins fit with this model (Knuepfer *et al*., 2014), and it remains to be established both how rhoptry sub‐structures are established and maintained and why different apicomplexan members have such varying rhoptry numbers (Sam‐Yellowe, 1996; Dubremetz, 2007) .

In addition to rhoptry ultrastructural detail, there is also a lack of appreciation regarding the potential for micronemal ultrastructure or the presence of subpopulations of these likely heterogeneous organelles (Healer *et al*., 2002; Kremer *et al*., 2013) and how proteins originating from different organelles meet to form multi‐meric complexes on the merzoite surface such as the interaction of apical membrane antigen (AMA)‐1 with the rhoptry neck (RON) complex (Alexander *et al*., 2005; Alexander *et al*., 2006). One model that would account for such complexes involves fusion events between certain micronemes and rhoptries at the neck that allows for their combined release; however, no experimental evidence for this model exists. Furthermore, the fact that rhoptry release can be uncoupled from micronemal release in *Toxoplasma* suggests the presence of independent secretion routes (Ravindran *et al*., 2009).

Apical organelle secretion preceding merozoite invasion creates a concentration gradient of invasion‐related proteins on the parasite surface, with each protein starting out most densely concentrated at the apical tip. As such, if the affinity of micronemal or rhoptry protein interactions with the erythrocyte surface exceeds affinity of the initial (perhaps MSP‐dependent) interaction then the merozoite will re‐orientate to its apex, without any additional energetic input (Dasgupta *et al*., 2014b). Although this has not been proven experimentally, in support of this model, merozoites have only a short window for host cell invasion, losing their re‐orientation ability and invasiveness within 3 min (Crick *et al*., 2014). Seen in this light, secreted proteins, diffusing through the merozoite surface would dissipate the concentration gradient leading to an inability to re‐orientate (Dasgupta *et al*., 2014b). Thus, re‐orientation may be largely a numbers game rather than active, and it is at this stage that commitment to invasion is likely established (Riglar *et al*., 2011). Investigating merozoite transmembrane protein diffusion dynamics using fluorescence recovery after photo‐bleaching could be very powerful for testing such hypotheses.

## A balanced interaction with the erythrocyte during invasion

Two major classes of secreted protein are defined as governing the ability to fulfil invasion of the erythrocyte and do so via alternative surface receptors. The two classes, hailing from micronemes and rhoptries respectively, are the erythrocyte binding‐like (or erythrocyte‐binding antigens (EBA) in *P*. *falciparum*) and reticulocyte binding‐like (RBL or Rh protein in *P*. *falciparum*) protein families (Paul *et al*., 2015). In *P*. *falciparum*, these proteins appear to work cooperatively (Lopaticki *et al*., 2011) and together define alternative routes for initiating the key steps of invasion proper. Variability across isolates in receptor‐dependence is well described (e.g. (Mensah‐Brown *et al*., 2015)), but it is not yet clear how EBA/RBL combinations define this variation. In *P*. *falciparum*, most members of the EBA proteins interact with erythrocyte glycophorin proteins, such as EBA175 to GPA or EBA140 to glycophorin C. Interaction with these (and other) core surface elements of the erythrocyte that link to its cytoskeleton – a hexagonal mesh underlying the lipid bilayer primarily consisting of spectrin and actin (Mohandas and Gallagher, 2008) – would therefore seem to be a key theme in invasion. Less is known about potential receptors for the Rh proteins, except for Rh4 that interacts with complement receptor 1 (CR1) (Spadafora *et al*., 2010; Tham *et al*., 2010) and Rh5 that binds basigin on the erythrocyte surface (Crosnier *et al*., 2011; Wright *et al*., 2014). However, given Rh5's markedly reduced size and that it has proven to be essential to all parasite lines tested (others Rh's appear to show some redundancy), it is not clear whether it should be considered as an independent functional class of invasion protein (Weiss *et al*., 2015). Binding of Rh proteins has been implicated in intracellular signalling within the parasite, possibly governing further release of key effectors of invasion (Engelberg *et al*., 2013; Gao *et al*., 2013). Whether these proteins play a mechanistic role in the physical process of invasion is, however, entirely unknown.

**Figure 3:**
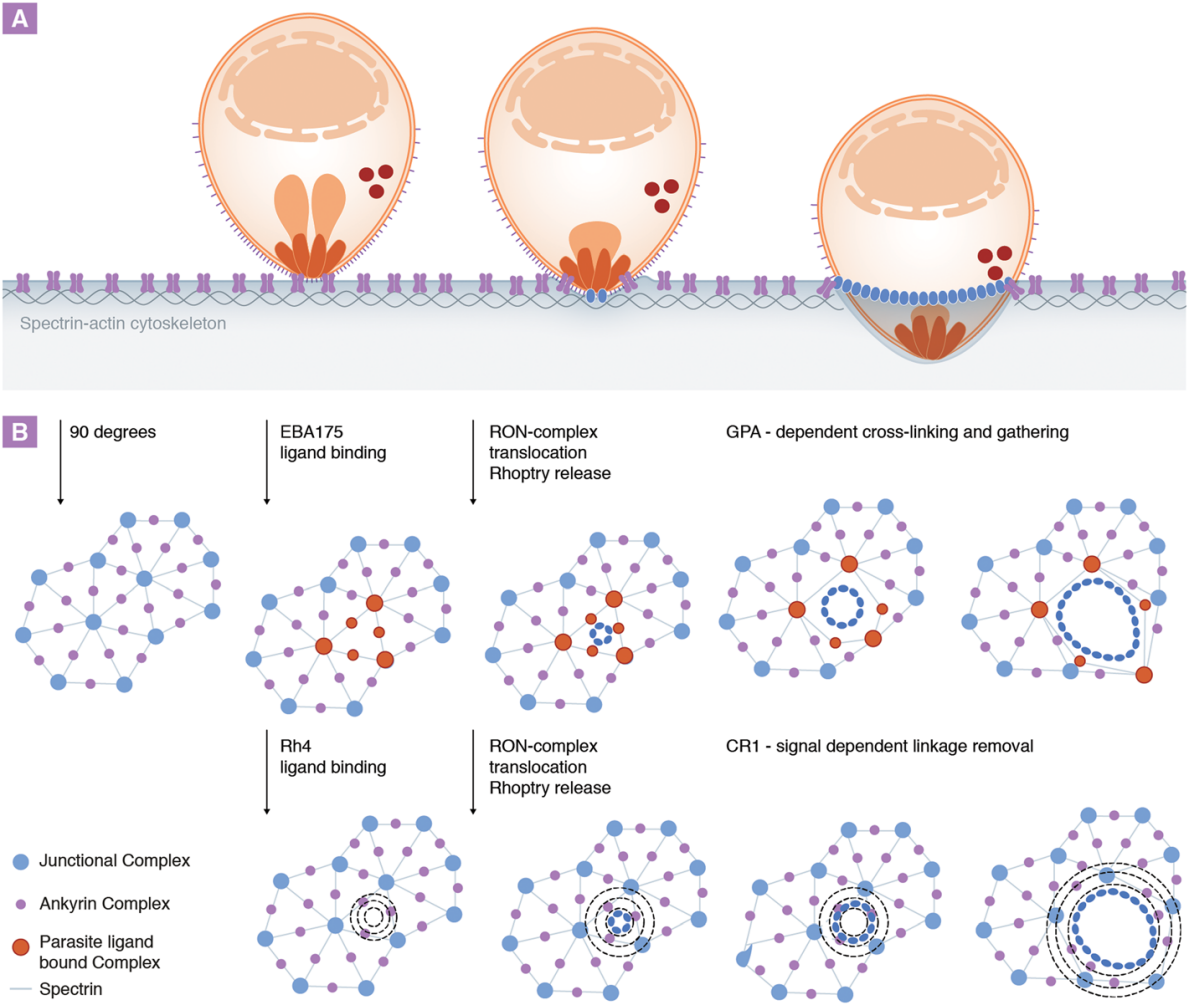
Model for *Plasmodium* merozoite invasion‐related protein and host receptor‐mediated cytoskeletal changes during invasion of erythrocytes. A. The erythrocyte actin–spectrin cytoskeleton (blue) is connected to the cell membrane via transmembrane protein (ankyrin and junctional) complexes but is cleared around the invading parasite via an unknown mechanism. B. Bird's eye view of the host cell cytoskeleton. Binding to GPA (red), which is contained within ankyrin and junctional protein complexes, is known to trigger increased cross‐linking of the cytoskeleton to GPA complexes. Tight attachment of parasite‐bound receptor complexes to the cytoskeleton via this route could therefore provide a structural anchor for invasion and/or ‘grip’ to pull open the spectrin mesh around the invading parasite (blue). In contrast, ligation of CR1 is known to cause phosphorylation of the cytoskeletal proteins spectrin and adducin (dotted circles), which are associated with dissociation of actin from the spectrin mesh junctions and increased membrane deformability. Thus, targeting this route could lead to cytoskeletal and mechanical changes that might provide the merozoite with an alternative mechanism of host cytoskeleton clearance at the entry site.

The erythrocyte cytoskeleton is tethered vertically to the membrane through protein complexes such as the junctional and ankyrin complex (Mohandas and Gallagher, 2008). Fluorescence recovery after photo‐bleaching and membrane diffusion experiments have shown that these connections are highly dynamic (van den Akker *et al*., 2010). As such, despite the cytoskeleton's role in stability and shape determination, erythrocyte deformability is unrestricted with the biochemical properties of spectrin allowing it to assemble into higher form oligomers for contraction or disassemble into dimers to allow extensive shape changes as required (An *et al*., 2002). These dynamics are of particular interest during invasion where the cytoskeleton must be cleared around the invading merozoite to allow it to enter (Fig. 3). How might this be achieved? Experimentally, binding of either complement molecule 3b (C3b) or specific antibodies to GPA (akin to EBA175 binding) has previously been shown to cause increased cross‐linking of the cytoskeleton to GPA‐containing protein complexes via the receptor's cytoplasmic tail. Under these conditions, this leads to a general decrease in erythrocyte deformability and stability (Chasis *et al*., 1988, Karnchanaphanurach *et al*., 2009). Intriguingly CD55, a glycosyl‐phosphatidylinositol‐linked surface protein recently identified as an essential host factor for merozoite invasion, also associates with immobilized GPA protein complexes via C3b, which results in its confinement with GPA and prevents autonomous complement‐mediated cell destruction (Karnchanaphanurach *et al*., 2009; Egan *et al*., 2015). It is not clear how such interactions would help invasion – particularly how increased rigidity would benefit the entering merozoite when the opposite might be expected. One possibility is that tighter cross‐linking of the erythrocyte membrane to the cytoskeleton provides increased anchorage for the parasite (perhaps by providing an anchor point for the tight junction (Besteiro *et al*., 2011)), which could then act as a traction point against which the parasite can push itself into the host cell. Alternatively, the GPA anchor could act as a confined boundary from which the spectrin–actin mesh could be stretched or opened up around the invading parasite (Gonzalez *et al*., 2009; Bichet *et al*., 2014). Either way, with so many invasion proteins binding to cytoskeleton‐related elements, the chance that their interactions with surface receptors fires off internal processes with the red cell, which might alter its overall biophysical architecture, is certainly attractive.

One primary caveat to understanding the potential role of some of these ligand–receptor interactions is that many of the core identified invasion‐related proteins are either non‐essential, or loss of their function can be compensated for by up‐regulation of alternative invasion ligands. For example, some *P*. *falciparum* strains can switch their reliance from the EBA175‐GPA sialic acid‐dependent interaction to a sialic acid‐independent pathway via Rh4 binding to CR1. Intriguingly, this alternative invasion pathway is also associated with receptor‐mediated alterations to the cytoskeleton, as CR1 binding triggers phosphorylation of spectrin and adducin. This change is associated with dissociation of actin from the spectrin network and, in contrast to the previously mentioned, increased membrane deformability (Glodek *et al*., 2010). Convergence on a common pathway (perhaps CD55 or Basigin dependent – which unlike GPA and CR1 appear to be essential for all *P*. *falciparum* strains) would now appear to be of key importance for sialic acid‐dependent and independent invasion. Basigin is a widely expressed multifunctional transmembrane protein implicated in inflammation and tumour invasion, but little is known with regard to its function or signalling mechanisms in the erythrocyte (Muramatsu and Miyauchi, 2003). Interestingly, CD55 is a receptor frequently used by a variety of viruses to enter host cells. In the epithelial cell line Caco‐2, virus binding to CD55 causes receptor clustering and activation of a signalling cascade involving the Src and Fyn kinases, the latter of which was found to be directly involved in cytoskeletal re‐organization and virus internalization (Coyne and Bergelson, 2006), themes reminiscent of the requirements for merozoite invasion. Thus, it may transpire that the merozoite has evolved to exploit several distinct routes to manipulate common erythrocyte cytoskeletal elements in a way that facilitates cell entry. It should be noted that the time frame in which host cell signalling cascades have to be initiated by the parasite to be meaningful for invasion is very narrow (in the matter of seconds) because of the speed of invasion. While it is known that CR1‐mediated cytoskeletal phosphorylation peaks at around 10 min following receptor binding using an antibody (Glodek *et al*., 2010), invasion is a very local event in which lower levels of phosphorylation might still be sufficient to prime the host cell. Importantly, it is now established that merozoite–erythrocyte receptor interactions do indeed induce specific cytoskeletal phosphorylation events in the erythrocyte at attachment (Zuccala and Satchwell, 2016). Regarding GPA‐dependent signalling, dynamics of its association with other cytoskeletal components have thus far only been explored on a limited timescale (Knowles *et al*., 1994).

A second caveat worth noting is that usage of erythrocyte receptors is not universal across *Plasmodium* species despite their ability to infect the same target erythrocyte (*Plasmodium knowlesi* and *Plasmodium vivax*, for example, are entirely reliant on the erythrocyte Duffy chemokine receptor for invasion of human erythrocytes – although *P*. *knowlesi* can invade rhesus erythrocytes independently of Duffy (Paul *et al*., 2015)). As such, either divergence between species defines alternative routes for initiating the invasion process or the core machinery (mechanistic process) of invasion may be independent of these initial parasite–surface interactions. A greater appreciation for receptor and cytoskeleton distribution and signalling processes during merozoite entry from each species is therefore clearly required to understand the significance of targeting the different surface receptors in invasion. Recent identification of both CD44 as another potential host protein required for efficient *P*. *falciparum* invasion may provide further avenues to explore the broader *Plasmodium*–erythrocyte question (Egan *et al*., 2015).

## Passage through the invasion aperture – structural nexus or boundary domain?

When binding to the erythrocyte surface with high‐affinity EBA or RBL proteins is blocked, release of rhoptry neck proteins is inhibited. This happens before the junction forms and suggests an ordering in the process from attachment orientation to formation of the tight, or moving, junction (Riglar *et al*., 2011). The junction is thought of as an anchoring structure through which the parasite drives itself into the nascent parasitophorous vacuole (Besteiro *et al*., 2011). One of its central architectural features is an interaction between the micronemal protein AMA1 and the RON complex of proteins from the rhoptry neck, secreted from their respective organelles into or onto the host cell surface. This molecular interaction is conserved across many apicomplexan parasites (Alexander *et al*., 2005; Alexander *et al*., 2006) and has been suggested to act as the bridging molecular link binding the parasite surface to both host cell and, internally, the parasite motor complex (Besteiro *et al*., 2011). Fitting with such a role, AMA1 has been shown to be critical for *Plasmodium* blood stage growth where blocking the interaction inhibits merozoite invasion (Richard *et al*., 2010; Srinivasan *et al*., 2011; Yap *et al*., 2014). Structural insights into AMA1 binding of RON2 (its partner molecule) certainly point to the potential for a very stable structure, capable of withstanding the mechanical stress of the motor (Tonkin *et al*., 2011; Vulliez‐Le Normand *et al*., 2012). However, the universality of the AMA1 requirement and its specific role during invasion is coming under increasing challenge. Junction formation and invasion in *Toxoplasma* and *Plasmodium* parasites can still occur when AMA1 has been deleted (reviewed in (Bargieri *et al*., 2014)). Intriguingly invasion still occurs through the RON‐ring structure in these AMA1 conditional‐knockout parasites. Is AMA1 therefore simply engaged with the junction but not a mechanical link between host cell and parasite? While these mutant parasites certainly invade host cells less efficiently than their control counterparts, the limiting factor appears to be reduced host cell attachment rather than blockage of cell entry (Bargieri *et al*., 2013). Thus even a complete mechanistic (or singular) functional role for AMA1 is currently left wanting.

As an alternative model, rather than seeing the junction as a structural component required for anchorage during invasion, it may instead serve as a point of demarcation, creating a biophysical and molecular boundary between the lipids and cytoskeletal elements directly underlying the site of invasion and the remainder of the host membrane (Dasgupta *et al*., 2014b). Secretion of rhoptry contents, including parasite‐derived lipids through this molecular seal into a segregated membrane area (Bannister *et al*., 1986) would then be expected to further decrease the local membrane tension. This would not only reduce the amount of force required by the merozoite to push itself into the host cell but also potentially induce local clearing of cytoskeletal elements (Dasgupta *et al*., 2014b). To explore this, it will be necessary to resolve the long‐standing debate about the origin of the parasitophorous vacuole and the contribution of host and parasite lipids to its formation (Lingelbach and Joiner, 1998).

## The final push for invasion

As the merozoite enters the host cell, the junction moves rearwards along the parasite driven by a substrate‐dependent gliding (Heintzelman, 2015). This is powered by an actin–myosin contractile system inside the parasite, which, according to current thinking, progresses using the force of the gliding motor (myosin A) pulling filamentous actin that transmits this force into rearward movement of surface‐secreted micronemal adhesion proteins – including members of the thrombospondin‐related anonymous protein or TRAP family. By linking intracellular force to such extracellular adhesins (Baum *et al*., 2006), the parasite is literally forced into the erythrocyte. It relies on a molecular bridge connecting the parasite actin–myosin motor to surface adhesins. Until recently, the glycolytic enzyme aldolase was thought to provide this role, binding with actin and interacting simultaneously with several merozoite surface adhesins including AMA1 and merozoite TRAP (Baum *et al*., 2006; Sheiner *et al*., 2010; Uchime *et al*., 2012). Although aldolase has proven to be an essential parasite protein, recent work in *Toxoplasma* showed that its critical function is limited to metabolism and not invasion (Shen and Sibley, 2014). Recent imaging data further suggest the merozoite TRAP extracellular domain is entirely absent during invasion (Riglar *et al*., 2015) compromising its role linking force to the erythrocyte surface (Baum *et al*., 2006). In *T*. *gondii*, conditional knockouts of some of the key actin–myosin motor components have also combined to entirely overhaul some of our core assumptions about apicomplexan host cell invasion in general (Meissner *et al*., 2013; Egarter *et al*., 2014). There is of course the possibility that there is redundancy within each protein family (Lamarque *et al*., 2014), but there is certainly an urgency to question our current understanding of the actin–myosin motor being the only force component required for apicomplexan invasion (Egarter *et al*., 2014). This could be a key area where again defining host contribution could be very important.

## Concluding remarks

With so many uncertainties remaining regarding the roles of most *Plasmodium* proteins functioning during invasion, we are still a distance away from a mechanistic understanding of erythrocyte invasion by the malaria parasite. Clearly, many of our long‐standing models for invasion require adjusting. This need not be a negative thing; one very positive element is that application of new technologies enables us for the first time to start challenging old dogmas towards overhauling not just *Plasmodium* invasion of the erythrocyte but potentially all apicomplexan host cell invasion. Of key importance, with so many of the core components now shown to be non‐essential or of unknown importance, reconsideration of the host cell, which could either through co‐option or manipulation contribute substantially towards lowering the energy requirements to achieve entry, deserves our focussed attention. For the blood stage malaria parasite, as we begin to appreciate how dynamic the erythrocyte really is, it is time to address both how the erythrocyte cytoskeleton and signalling networks therein respond to merozoite invasion and how these might work in favour of entry. Finally, as the temptation to describe further effectors of invasion expands, it is the view of these authors that rather than accept a functional definition of invasion proteins in terms of essentiality (as in a statistical drop in invasion efficiency) or the ability of a protein to bind a defined receptor, functional classification must move towards assessing what proteins mechanistically do during invasion if we are to wholly understand the process. Combining these concepts, shifting our world view to a balanced interaction between parasite and host underpinning infection, we may discover new, and innovative, ways to stop the process as a way to treat or prevent disease.
